# Engineering of acetyl-CoA metabolism for the improved production of polyhydroxybutyrate in *Saccharomyces cerevisiae*

**DOI:** 10.1186/2191-0855-2-52

**Published:** 2012-09-25

**Authors:** Kanokarn Kocharin, Yun Chen, Verena Siewers, Jens Nielsen

**Affiliations:** 1 Department of Chemical and Biological Engineering, Chalmers University of Technology, Kemivägen 10, SE-412 96, Göteborg, Sweden

**Keywords:** Polyhydroxybutyrate, Acetyl coenzyme A, *Saccharomyces cerevisiae*, Pathway engineering

## Abstract

Through metabolic engineering microorganisms can be engineered to produce new products and further produce these with higher yield and productivities. Here, we expressed the bacterial polyhydroxybutyrate (PHB) pathway in the yeast *Saccharomyces cerevisiae* and we further evaluated the effect of engineering the formation of acetyl coenzyme A (acetyl-CoA), an intermediate of the central carbon metabolism and precursor of the PHB pathway, on heterologous PHB production by yeast. We engineered the acetyl-CoA metabolism by co-transformation of a plasmid containing genes for native *S. cerevisiae* alcohol dehydrogenase (*ADH2*), acetaldehyde dehydrogenase (*ALD6*), acetyl-CoA acetyltransferase (*ERG10*) and a *Salmonella enterica* acetyl-CoA synthetase variant (*acs*^L641P^), resulting in acetoacetyl-CoA overproduction, together with a plasmid containing the PHB pathway genes coding for acetyl-CoA acetyltransferase (*phaA),* NADPH-linked acetoacetyl-CoA reductase *(phaB)* and poly(3-hydroxybutyrate) polymerase (*phaC)* from *Ralstonia eutropha* H16. Introduction of the acetyl-CoA plasmid together with the PHB plasmid, improved the productivity of PHB more than 16 times compared to the reference strain used in this study, as well as it reduced the specific product formation of side products.

## Introduction

Poly-(R)-3-hydroxybutyrate (PHB) is the most common type of polyhydroxyalkanoates (PHAs) synthesized and accumulated by microorganisms like *Ralstonia eutropha* (also known as *Cupriavidus necator, Wautersia eutropha*, *Alcaligenes eutrophus*), *Bacillus megaterium* or *Pseudomonas* sp. as carbon and energy storage material in response to conditions of physiological stress (Steinbuchel et al. 1993). Biodegradable PHB is a linear polyester consisting solely of the stereospecific monomer, (R)-3-hydroxybutyric acid. It belongs to the group of short chain length PHAs consisting of C_3_-C_5_ hydroxyacid monomers. (Hankermeyer and Tjeerdema 1999; Melchiors et al. 1994). The biosynthesis pathway of PHB involves three enzymes and their sequential reactions (Breuer et al. 2002; Carlson et al. 2002; Steinbüchel 2001; Steinbüchel and Hein 2001). The first enzyme of the pathway is acetyl-CoA *C*-acetyltransferase [EC 2.3.1.9], encoded by *phaA,* which catalyzes the condensation of two acetyl-CoA molecules to form acetoacetyl-CoA (Peoples and Sinskey 1989b). The next step is the reduction of acetoacetyl-CoA to (R)-3-hydroxybutyryl-CoA, which is catalyzed by NADPH-dependent acetoacetyl-CoA reductase [EC 1.1.1.36] encoded by *phaB* (Peoples and Sinskey 1989b)*.* Finally, PHA synthase [EC 2.3.1.-] encoded by *phaC*, catalyzes the polymerization of (R)-3-hydroxybutyryl-CoA monomers to PHB (Peoples and Sinskey 1989a).

The natural PHB producers like *R. eutropha*, *Bacillus megaterium* or *Pseudomonas sp.* are known to produce and accumulate PHB as a storage compound in response to nutrient imbalance caused by growth under conditions of carbon source excess but limitation in other essential nutrients (Steinbüchel and Hein 2001; Trotsenko and Belova 2000). Instead of employing the natural PHB producers, which can depolymerize PHB and use it as a secondary energy source, metabolic engineering can be used to transfer the PHB biosynthetic pathway to alternative hosts that may have advantage over the natural PHB producers, in particular the lack of enzymes for PHB depolymerization (Uchino and Saito 2006). Furthermore, by transferring to alternative hosts one may take advantage of a range of technologies developed for general platform cell factories like *Escherichia coli* and *Saccharomyces cerevisiae*. Thus, there have been a range of studies where PHB production has been evaluated in *E. coli* and further metabolic engineering has been carried out with the objective to improve the productivity. In a metabolic and kinetic study, a recombinant *E. coli* strain producing PHB was examined and compared to the native PHB producer, *R. eutropha*, and this study revealed that the PHB flux was highly sensitive to the acetyl-CoA/CoA ratio, the total acetyl-CoA plus CoA concentration and pH (van Wegen et al. 2001). In recombinant *E. coli*, a mutation in *arcA* encoding a protein that regulates aerobic respiration under microaerobic conditions resulted in higher amounts of PHB accumulated in the cell (Nikel et al. 2006). Low-agitation conditions had a positive effect on PHB synthesis from glycerol in recombinant *E. coli* carrying the *phaCAB* operon and *phaP* encoding a granule-associated protein (phasin) (de Almeida et al. 2010). Several studies also attempted to synthesize PHB in plants according to the aim to produce high amounts of PHB at lower costs compared to microbial fermentation, particularly in plant plastids where the biosynthesis of fatty acids from acetyl-CoA occurs (Bohmert et al. 2000; Nawrath et al. 1994; Petrasovits et al. 2012). However, the growth of some of these transgenic plants was inhibited, possibly due to the metabolic burden of PHB synthesis (Poirier et al. 1992).

The benefits of using S. cerevisiae as a model for producing PHB is that the molecular machinery of *S. cerevisiae* is well studied and as the most widely used eukaryal microorganism for industrial production of fuels and chemicals it is an attractive cell factory platform. Furthermore, its genome has been very well characterized and genome scale metabolic models have been reconstructe*d* (Goffeau 2000; Nookaew et al. 2008). For these reasons, several attempts have been made to evaluate *S. cerevisiae* as a cell factory for PHB production (Breuer et al. 2002; Dimster-Denk and Rine 1996; Leaf et al. 1996; Marchesini et al. 2003; Zhang et al. 2006). Synthesis of PHB in *S. cerevisiae* has initially been demonstrated by expressing only bacterial polyhydroxybutyrate synthase (Leaf et al. 1996). This PHB synthesis approach is successful because of the activity of native thiolase and reductase enzymes involved in the synthesis of D-3-hydroxybutyryl-CoA in *S. cerevisiae.* However, the yield obtained in this study was very low when compared with the expression of all three genes of the PHB biosynthesis pathway (Breuer et al. 2002; Carlson et al. 2002; Carlson and Srienc 2006). Thiolase enzymes in *S. cerevisiae* exist and function in three different compartments, in mitochondria and peroxisomes for fatty acid β-oxidation and in the cytoplasm for the mevalonate pathway (Hiser et al. 1994). However, only the cytoplasmic thiolase participates in PHB biosynthesis. Many approaches have been followed to improve the production of PHB in yeast. Srienc and co-workers performed elementary mode analysis of a *S. cerevisiae* containing the PHB synthesis pathway in order to identify new metabolic engineering targets (Carlson et al. 2002). The analysis suggested that the introduction of the ATP citrate-lyase reaction and the transhydrogenase reaction can improve the theoretical PHB carbon yield (Carlson et al. 2002). Acetyl-CoA serves as the precursor for the PHB biosynthesis pathway and increasing the availability of acetyl-CoA was proposed to improve PHB production (Carlson and Srienc 2006; Suzuki et al. 2002). However, using enzyme inhibitors to reduce its consumption by other pathways or feeding of the substrate during cultivation would result in increasing production costs and may not be feasible for industrial applications. Here, we demonstrated metabolic pathway engineering by co-transformation of a plasmid containing the PHB biosynthesis pathway and an acetyl-coenzyme A (acetyl-CoA) boost plasmid designated to improve the availability of cytoplasmic acetyl-CoA and hereby improve the productivity of PHB in *S. cerevisiae*.

## Materials and methods

### Strains, media, and culture conditions

Plasmids were maintained and propagated in *E. coli* DH5α. The preparation of competent *E. coli* cells and their transformation were performed according to standard protocols (Sambrook and Russell 2006). Lysogeny broth (LB) medium was used for routine culturing of *E. coli* (Bertani 1951) and 80 mg L^-1^ ampicillin was added to LB when needed. *S. cerevisiae* strain CEN.PK113-11C (*MAT*a *SUC2 MAL28*^c^*ura352 his3-Δ1;* provided by P. Kötter, Frankfurt, Germany) was used as the background strain for evaluation of the polyhydroxybutyrate pathway. Plasmid containing yeast strains were selected on synthetic dextrose (SD) medium, prepared with 6.7 g L^-1^ yeast nitrogen base without amino acids (YNB-AA) (Formedium, Hunstanton, UK) and 20 g L^-1^ glucose with complete supplement mixture (CSM) lacking uracil and/or histidine (Formedium) where appropriate.

### Plasmid construction and yeast transformation

**Figure 1:**
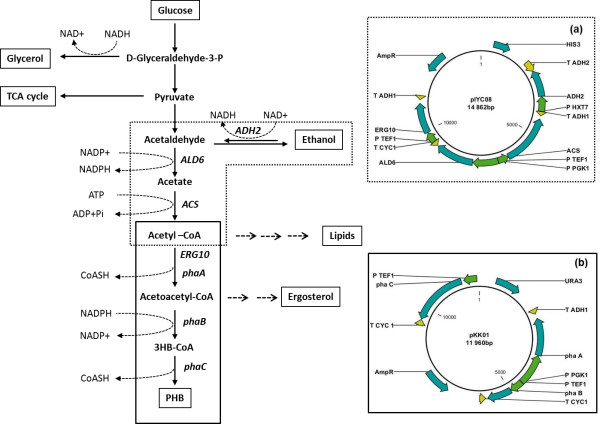
**Schematic pathway and plasmid maps for polyhydroxybutyrate production in*S. cerevisiae*.** (**a**) Acetyl-CoA boost plasmid (pIYC08) containing *ADH2*: alcohol dehydrogenase, *ALD6*: aldehyde dehydrogenase, *ACS*: acetyl-CoA synthetase variant and *ERG10*: acetyl-CoA acetyltransferase. (**b**) PHB plasmid (pKK01) containing PHB genes from *R. eutropha*, *phaA*: acetyl-CoA acetyltransferase, *phaB*: NADPH-linked acetoacetyl coenzyme A (acetyl-CoA) reductase and *phaC*: poly(3-hydroxybutyrate) polymerase. P and T in the plasmid map represent promoter and terminator, respectively.

The detailed construction of pIYC04 as a background plasmid and pIYC08 as acetyl-CoA boost plasmid is described by Chen et al. (Chen et al. 2012). The primers used for plasmid construction are listed in Table 1. The PHB biosynthesis pathway was introduced into CEN.PK 113-11C by using another multi-copy plasmid based on pSP-GM2 containing a P_*TEF1*_-P_*PGK1*_ bidirectional promoter (Partow et al. 2010). The PHB biosynthesis pathway genes *phaA*, *phaB* and *phaC* were synthesized based on the genes from *R. eutropha* H16 and codon optimized for expression in *S. cerevisiae* by DNA 2.0 (Menlo Park, CA, USA). *PhaA* was cloned into pSP-GM2 into the *Spe*I/*Sac*I sites between the *PGK1* promoter and the *ADH1* terminator. Then, *PhaB* was cloned into *the BamH*I*/Sal*I sites between the *TEF1* promoter and the *CYC1* terminator of the same vector to yield pSP-GM2-phaAB. *PhaC* was cloned into the MCS of pSP-GM2 vector the *TEF1* promoter and the *CYC1* terminator. The fragment of *phaC* together with the *TEF1* promoter and the CYC1 terminator was amplified using primer 1 and 2 and ligated into pSP-GM2-phaAB using the *Mfe*I restriction site resulting in pKK01. The direction of *phaC* insertion was confirmed by colony PCR with primer 3 and 4. Yeast transformation was performed by using the lithium acetate/single-stranded carrier DNA/ polyethylene glycol method (Gietz and Woods 2002). Strain SCKK005 was constructed by transforming plasmids pKK01 and pIYC04 into strain CEN.PK113-11C. Plasmids pKK01 and pIYC08 were co-transformed into strain CEN.PK113-11C for the construction of SCKK006. Strain SCKK009 and SCKK010 were constructed by co-transformation of plasmids pKK01 and pIYC08 into SIYC32 and SCIYC33, respectively. The construction of the *cit2Δ* strain (SCIYC32) and the *mls1Δ* strain (SCIYC33) are described by Chen et al. (Chen et al. 2012). Strains used in this study are summarized in Table 2. The metabolic pathway and plasmid maps are illustrated in Figure 1. 

**Table 1:** Oligonucleotides used in this study

**No.**	**Sequence (5' -3')**
1	TACAATTGCTATTATTATCCTGCTCAGTGGTACTT
2	TCCAATTGTCAGTGAGCGAGGAAGCGGAAGAG
3	TTCGTTCTTCCTTCTGTTCGGAGATTAC
4	GGAACAGGAGTATTGCCTTTCAAGTAGTTATC

Restriction sites are underlined.

**Table 2:** Yeast strains and plasmids used in this study

**Strain**	**Genotype or relevant feature(s)**	**Plasmid**	**Source**
CEN.PK 113-11C	*MAT*a *SUC2 MAL2*-*8*^c^*ura3*-*52 his3-Δ1*	-	P. Kötter^a^-
SCKK005	*MAT*a *SUC2 MAL2*-*8*^c^*ura3*-*52 his3-Δ1*	pIYC04/pKK01	This study
SCKK006	*MAT*a *SUC2 MAL2*-*8*^c^*ura3*-*52 his3-Δ1*	pIYC08/pKK01	This study
SCIYC32	*MAT*a *SUC2 MAL2*-*8*^c^*ura3*-*52 his3-Δ1 cit2Δ*	-	(Chen et al. 2012)
SCIYC33	*MAT*a *SUC2 MAL2*-*8*^c^*ura3*-*52 his3-Δ1 mls1Δ*	-	(Chen et al. 2012)
SCKK009	*MAT*a *SUC2 MAL2*-*8*^c^*ura3*-*52 his3-Δ1 cit2Δ*	pIYC08/pKK01	This study
SCKK010	*MAT*a *SUC2 MAL2*-*8*^c^*ura3*-*52 his3-Δ1 mls1Δ*	pIYC08/pKK01	This study

^a^ Institute of Microbiology, J.W. Goethe Universität, Frankfurt, Germany.

### Shake flask cultivation

The pre-cultures for shake flask cultivations were prepared by inoculation of 5 mL modified minimal medium in a 14 mL culture tube and grown at 30°C and 180. The modified minimal medium for shake flask cultivations was prepared as follows: 5 g⋅L^-1^ (NH_4_)_2_SO_4_; 3 g⋅L^-1^ KH_2_PO_4;_ 0.5 g⋅L^-1^ MgSO_4_⋅7H_2_O; 1 mL⋅L^-1^ of trace metal solution and vitamin solution (see below) with an initial pH of 6.5. 45 mL of defined minimal medium in a 100 mL unbaffled flask were inoculated with an amount of pre-culture that resulted in a final optical density of 0.02 at 600 nm (OD_600_). The culture was grown at 30°C with 180 rpm in an orbital shaking incubator and samples were taken at 40, 80 and 120 h to determine PHB production.

### Bioreactor cultivation

PHB production was evaluated in defined minimal media with the following composition: 5 g⋅L^-1^ (NH_4_)_2_SO_4_; 3 g L^-1^ KH_2_PO_4_; 0.5 g L^-1^ MgSO_4_⋅7H_2_O; 1 mL L^-1^ trace metal solution (pH 4.0: 15.0 g L^-1^ EDTA (sodium salt); 0.45 g L^-1^ ZnSO_4_⋅7H_2_O; 1 g L^-1^ MnCl_2_⋅2H_2_O; 0.3 g L^-1^ CoCl_2_⋅6H_2_O; 0.3 g L^-1^ CuSO_4_⋅5H_2_O; 0.4 g L^-1^ Na_2_MoO_4_⋅2H_2_O; 0.45 g L^-1^ CaCl_2_⋅2H_2_O; 0.3 g L^-1^ FeSO_4_⋅7H_2_O; 0.1 g L^-1^ H_3_BO_3_, and 0.10 g L^-1^ KI). The pH was adjusted to 5 by adding 2 M KOH and autoclaved separately from the carbon source solution. Glucose was added at a concentration of 20 g L^-1^. Vitamin solution (pH 6.5: 0.05 g L^-1^ biotin; 0.2 g L^-1^ ρ-amino benzoic acid; 1 g L^-1^ nicotinic acid; 1 g L^-1^ Ca-pantothenate; 1 g L^-1^ pyridoxine-HCl; 1 g L^-1^ thiamine-HCl and 25 g L^-1^ myo-inositol) was filter sterilized and aseptically added to the medium after autoclaving at the concentration of 1 mL L^-1^. Batch cultivations were carried out in 1.2 L bioreactors. The pre-cultures were prepared using the same culture conditions as for shake flask cultivation. 700 mL of defined minimal medium were inoculated with an amount of pre-culture that resulted in a final OD_600_ of 0.02. The temperature was kept at 30°C and the pH was adjusted to 5.00 ± 0.05 using 2 M KOH.

### Analytical methods

Culture samples of 10 mL volume were centrifuged at 5,311 × g and 4°C for 5 min and the pellets were washed once with distilled water and centrifuged at 14,000 × g for 1 min. To lyophilize the biomass, the recovered cell pellet was immediately frozen by immersion in liquid nitrogen followed by lyophilization under vacuum (Christ Alpha 2–4 LSC, Shropshire, UK). The cell dry weight was determined and the pellet kept at 4°C for further analysis.

Metabolites including glucose, ethanol, glycerol, and acetate were quantified in the culture supernatant using an Ultimate 3000 HPLC (Dionex, Sunnyvale, CA, USA) equipped with an Aminex HPX 87 H ion exclusion column (300 mm × 7.8 mm, Bio-Rad Laboratories, Hercules, CA, USA) which was operated at 45°C and a flow rate of 0.6 mL min^-1^ of 5 mM H_2_SO_4_ using a refractive index detector and UV detector for analysis of sugars and organic acids, respectively.

PHB was analyzed as described previously (Karr et al. 1983; Tyo et al. 2006). 10–20 mg of dried cells were weighed and boiled in 1 mL of concentrated sulfuric acid for 60 min and then diluted with 4 mL of 14 mM H_2_SO_4_. Samples were centrifuged (15 min, 16,000 × g) to remove cell debris, and the supernatant was analyzed using an Ultimate 3000 HPLC (Dionex) equipped with an Aminex HPX-87 H ion exclusion column (300 × 7.8 mm; Bio-Rad Laboratories) and UV detector. Commercially available PHB (Sigma-Aldrich, St. Louis, MO), processed in parallel with the samples, was used as a standard. The HPLC was operated at 60°C and a flow rate of 0.6 mL min^-1^ of 5 mM H_2_SO_4_.

The total lipid extraction method was adapted from Bligh and Dyer (Bligh and Dyer 1959). Briefly, 15 mg of freeze-dried cell pellets were treated with 1 unit μL^-1^ zymolyase in 1 mL digestion buffer (1.2 M glycerol, 100 mM sodium thioglycolate, 50 mM Tris-sulfate, pH 7.5) at 37°C for 15 min, followed by centrifugation at 3000 rpm for 3 min. The mixture of collected spheroplasts was spiked with cholesterol as internal standard, 7 ml of chloroform:methanol (2:1, v/v) were added and shaken vigorously at room temperature and 500 rpm for 30 min. 1.7 mL of 0.73% NaCl was added to the mixture prior centrifugation at 3500 rpm for 5 min at 4°C. The lower (organic) phase was collected and the upper phase was re-extracted with 5 mL of chloroform-methanol (85:15 v/v). The lower (organic) phase was collected and pooled with the previous organic fraction and kept at −20°C for further HPLC analysis.

Lipid separation and quantification were performed using the method modified from Silversand and Haux (Silversand and Haux 1997). Lipid separation was accomplished using an Ultimate 3000 HPLC (Dionex) equipped with a Corona charged aerosol detector (CAD) (Dionex) connected with nitrogen gas at 35 psi gas pressure. A 20 μL volume of each sample was injected into a Luna 5 μm HILIC 200 Å 250 × 4.6 mm LC column (Phenomenex, Torrance, CA). The flow-rate was 0.8 mL⋅min^-1^ and the column temperature was kept at 25°C during all runs. A gradient flow with 3 solvents (solvent A, 99:1 by vol. of hexane/acetic acid, solvent B 70:29:1, by vol. of hexane/isopropanol/acetic acid, solvent C 85:14:1, by vol. of isopropanol/water/acetic acid) was applied for the separation of lipids. Triethylamine (0.08%) was added to solvent B and solvent C for better separation. The HPLC system was equilibrated with 99% solvent A. The gradient profile started from 3% solvent B at 7 min, reached 10% solvent B in 1 min and ended at 100% solvent B in 15 min, while solvent C was kept at 0% from 0 min to 15 min. This gradient profile is used for separation of triacylglycerol (TAG), free fatty acids, cholesterol (as internal standard) and ergosterol. After 15 min, solvent A was kept at 0% until 35 min. Meanwhile, an increase of solvent C to 65% from 15 min to 32 min was applied for separation of phospholipids (phosphatidylethanolamine, phosphatidylinositol, phosphatidylcholine, phosphatidylserine, phosphatidic acid). After that, solvent C was decreased to 0% from 32 min to 35 min and 100% solvent B was applied to the system. After 40 min, 99% solvent A was run for 5 min in order to equilibrate and stabilize the system for the measurement of the next sample. A logarithmic plot of peak area versus the concentration of each lipid standard was used to generate a calibration curve. The slope determined from the log-log plot was further used for lipid quantification (Nair and Werling 2009).

## Results

### Characterization of PHB-producing *S. cerevisiae*

**Figure 2:**
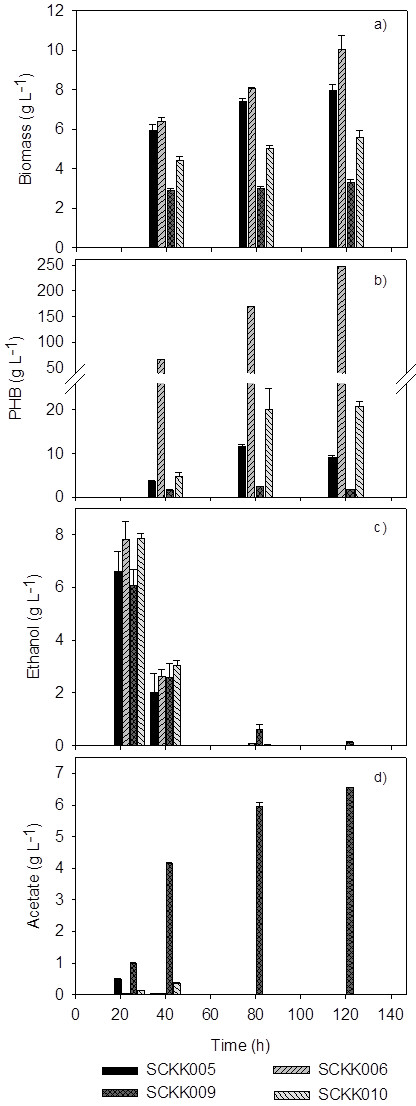
**Measurements of biomass and PHB from shake flask cultivations in a modified minimal medium with 20 g L**^**-1**^**glucose as carbon source.** Strain SCKK005 harbors an empty plasmid (pIYC04) and the PHB plasmid (pKK01), strain SCKK006 harbors an acetyl-CoA boost plasmid (pIYC08) and the PHB plasmid (pKK01), SCKK009 and SCKK010 harbor pIYC08 and pKK01 and carry a *CIT2* and *MLS1* deletion, respectively.

The engineered *S. cerevisiae* strains were preliminary studied for growth and PHB production in shake flasks as shown in Figure 2. Co-expression of beta-ketothiolase, acetoaetyl-CoA reductase and PHA synthase results in PHB accumulation in *S. cerevisiae* as observed in previous studies (Breuer et al. 2002; Carlson et al. 2002; Carlson and Srienc 2006). In this study, the 3 genes involved in the PHB pathway were expressed from a single vector in order to avoid the heterogeneity of plasmid distribution. *S. cerevisiae* carrying the PHB plasmid and an empty *HIS3* plasmid (strain SCKK005) and a strain carrying the PHB plasmid and the acetyl-CoA boost plasmid (SCKK006) were characterized and evaluated for the productivity of PHB. The acetyl-CoA boost plasmid contains 4 genes, *ADH2*, *ALD6*, *acs*^L641P^ and *ERG10*, involved in channeling carbon from ethanol to acetyl-CoA. In the acetyl-CoA boost plasmid, *ALD6*, *acs*^L641P^ and *ERG10* are controlled by constitutive promoters, P_*TEF1*_ and P_*PGK1*_, respectively, while *ADH2* is under control of the P_*HXT7*_ promoter which is strongly de-repressed under glucose depletion (Partow et al. 2010; Reifenberger et al. 1995; Sedlak and Ho 2004). 

*CIT2* encoding peroxisomal citrate synthase catalyzes the conversion of oxaloacetate and acetyl-CoA to citrate and plays role in acetate metabolism. *MLS1* encoding malate synthase catalyzes the conversion of glyoxylate and acetyl-CoA to malate in the glyoxylate shunt. Deletion of *CIT2* and *MLS1* affect the integrity of the glyoxylate shunt and will therefore reduce the drain of acetyl-CoA through this pathway and hereby possibly affect the availability of cytosolic acetyl-CoA. Thus, the effect of *CIT2* and *MLS1* deletion on PHB production was also investigated. The biomass yield of *cit2Δ* (SCKK009) was less than that of *mls1Δ* (SCKK010), and the biomass yield for both the deletion strains were lower than those of the non-deletion reference strain without the acetyl-CoA plasmid (SCKK005) and the non-deletion strain with the acetyl-CoA plasmid (SCKK006) (see Figure 2a). The biomass yield of the deletion strains, SCKK009 and SCKK010, was lower than that of non-deletion strains, SCKK005 and SCKK006, due to the impaired C_2_ carbon utilization. The recombinant strain with both the acetyl-CoA boost plasmid and the PHB plasmid (SCKK006) produced an 18 times higher final concentration of PHB (at 120 h) compared to the reference strain, SCKK005 (Figure 2b). Besides that, the amount of accumulated PHB in SCKK009 and SCKK010 was less than SCKK006. Although, *mls1Δ* (SCKK010) gave lower biomass yield than the reference, it gave higher PHB titer compare to the non-deletion reference strain (SCKK005) when the deletion strain carried the acetyl-CoA plasmid. This result showed the combined effect of *mls1Δ* together with the utilization of acetyl-CoA plasmid on the availability of acetyl-CoA and the influence of the deletion on PHB production. The consumption rate of ethanol in the deletion strains, especially in *cit2Δ* (SCKK009), was slower than for the non-deletion strain as the residual amount of ethanol was detected after 80 h of cultivation (Figure 2c). In addition, more than 6.5 g L^-1^ of acetate was detected in the medium after 120 of fermentation of SCKK009, which is clear evidence for the impaired C_2_ metabolism. Due to the negative impact of *CIT2* and *MLS1* deletion on growth and PHB production, the recombinant strains SCKK005 and SCKK006 without gene deletions were selected for further characterization in bioreactors.

**Figure 3:**
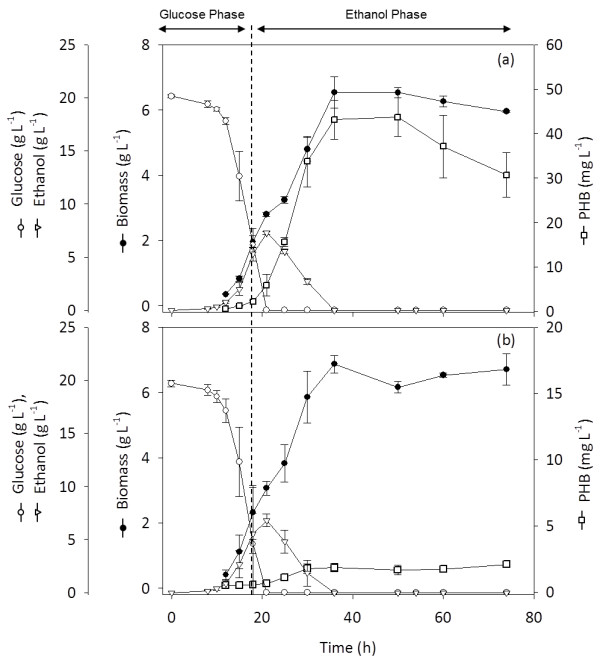
**Fermentation profile of*S. cerevisiae*producing PHB in aerobic batch bioreactor cultivation using a chemically defined medium with 20 g L**^**-1**^**glucose as carbon source.** (**a**) SCKK006: *S. cerevisiae* harboring an acetyl-CoA boost plasmid (pIYC08) and the PHB plasmid (pKK01). (**b**) SCKK005: *S. cerevisiae* harboring an empty plasmid (pIYC04) and the PHB plasmid (pKK01).

Kinetic studies of the PHB-producing *S. cerevisiae* strains were carried out in aerobic bioreactor cultivation. The production and accumulation of PHB coincided with the depletion of glucose and the increase in ethanol concentration during the glucose consumption phase. SCKK005 and SCKK006 demonstrated a similar growth profile as shown in Figure 3. Kinetic parameters and yields on glucose and ethanol are summarized in Table 3. There was no significant difference in the maximum specific growth rates of SCKK005 and SCKK006, which were 0.27 ± 0.02 h^-1^ and 0.28 ± 0.00 h^-1^, respectively. The glucose consumption rate of SCKK006 was higher than that of SCKK005. However, a slightly lower biomass yield on glucose of SCKK006 was observed. The strain carrying the acetyl-CoA boost plasmid showed the capability to increase the carbon flux from ethanol to the PHB pathway as the PHB yield on ethanol in SCKK006 was significantly higher than that of SCKK005 In the ethanol phase, the PHB yield on ethanol in SCKK006 was 6.09 ± 1.44 mg (g EtOH)^-1^, which was approximately 25-fold higher than for SCKK005 that had a yield of 0.22 ± 0.04 mg (g EtOH)^-1^. The maximum PHB titer detected during the ethanol phase in SCKK005 was 1.85 mg⋅L^-1^ while SCKK006, which contained both the PHB biosynthesis and the acetyl-CoA boost plasmid, reached a titer of 43.11 mg⋅L^-1^ after 36 h of batch fermentation. In SCKK005, the PHB level remained at the same concentration until the end of fermentation while the PHB titer in SCKK006 tended to decrease after 50 h. 

**Table 3:** Yields and kinetic parameters obtained from batch cultivations

	**Unit**	**SCKK005**	**SCKK006**
Maximum specific growth rate	h^-1^	0.27 ± 0.02	0.28 ± 0.00
Glucose consumption rate	g (g DW h)^-1^	1.80 ± 0.09	2.24 ± 0.33
Biomass yield on glucose	g (g glc)^-1^	0.15 ± 0.01	0.13 ± 0.02
Ethanol yield on glucose	g (g glc)^-1^	0.35 ± 0.05	0.35 ± 0.07
Glycerol yield on glucose^*^	g (g glc)^-1^	0.05 ± 0.00	0.07 ± 0.00
Acetate yield on glucose^*^	g (g glc)^-1^	0.02 ± 0.00	0
PHB yield on glucose^*^	mg (g glc)^-1^	0.02 ± 0.01	0.13 ± 0.02
Biomass yield on ethanol	g (g EtOH)^-1^	0.46 ± 0.27	0.45 ± 0.08
PHB yield on ethanol^*^	mg (g EtOH)^-1^	0.22 ± 0.04	6.09 ± 1.44

The values were calculated from at least triplicate fermentations (n ≥ 3) and represent as mean ± SD.

^*^ The values are significantly difference at *p*-value ≤ 0.05.

### Comparison of specific productivities in pathway engineered strains

**Figure 4:**
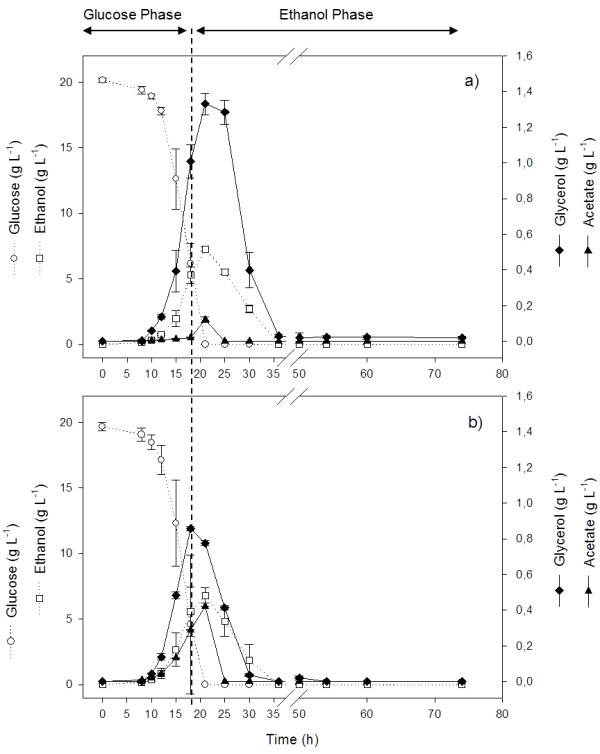
**Time profile of glucose consumption and ethanol, glycerol and acetate formation during batch bioreactor cultivation of a) SCKK006 and b) SCKK005 in a chemically define minimal medium with 20 g L**^**-1**^**glucose as carbon source.** SCKK006: *S. cerevisiae* harboring an acetyl-CoA boost plasmid (pIYC08) and the PHB plasmid (pKK01). and SCKK005: *S. cerevisiae* harboring an empty plasmid (pIYC04) and the PHB plasmid (pKK01), respectively.

The effect of the acetyl-CoA boost plasmid was clearly seen during growth in the glucose phase when SCKK006 started to produce and accumulate PHB with a yield of PHB on glucose of 0.13 ± 0.02 mg (g glc)_,_^-1^ higher than that of SCKK005 as previously shown in Table 3. Figure 4 shows the consumption of glucose and the formation of ethanol, glycerol and acetate measured during growth in an aerobic batch bioreactor. A maximum glycerol concentration of 1.33 ± 0.06 g⋅L^-1^ was observed in SCKK006, while a lower glycerol concentration, 0.86 ± 0.01 g⋅L^-1^, was observed in SCKK005. This result indicates the influence of *ADH2* overexpression in order to convert ethanol to acetaldehyde thus resulting in a small increase in NADH production, which triggers the formation of glycerol for redox balancing in the cytosol (Bakker et al. 2001). On the other hand, the maximum acetate concentration of 0.42 ± 0 g⋅L^-1^ detected in SCKK005 was 3 times higher than the acetate concentration of 0.12 ± 0.01 g⋅L^-1^ detected in SCKK006. Therefore, the different concentrations of glycerol and acetate detected during growth on glucose are most likely a consequence of introducing the acetyl-CoA boost plasmid in the PHB producing strain. 

**Figure 5:**
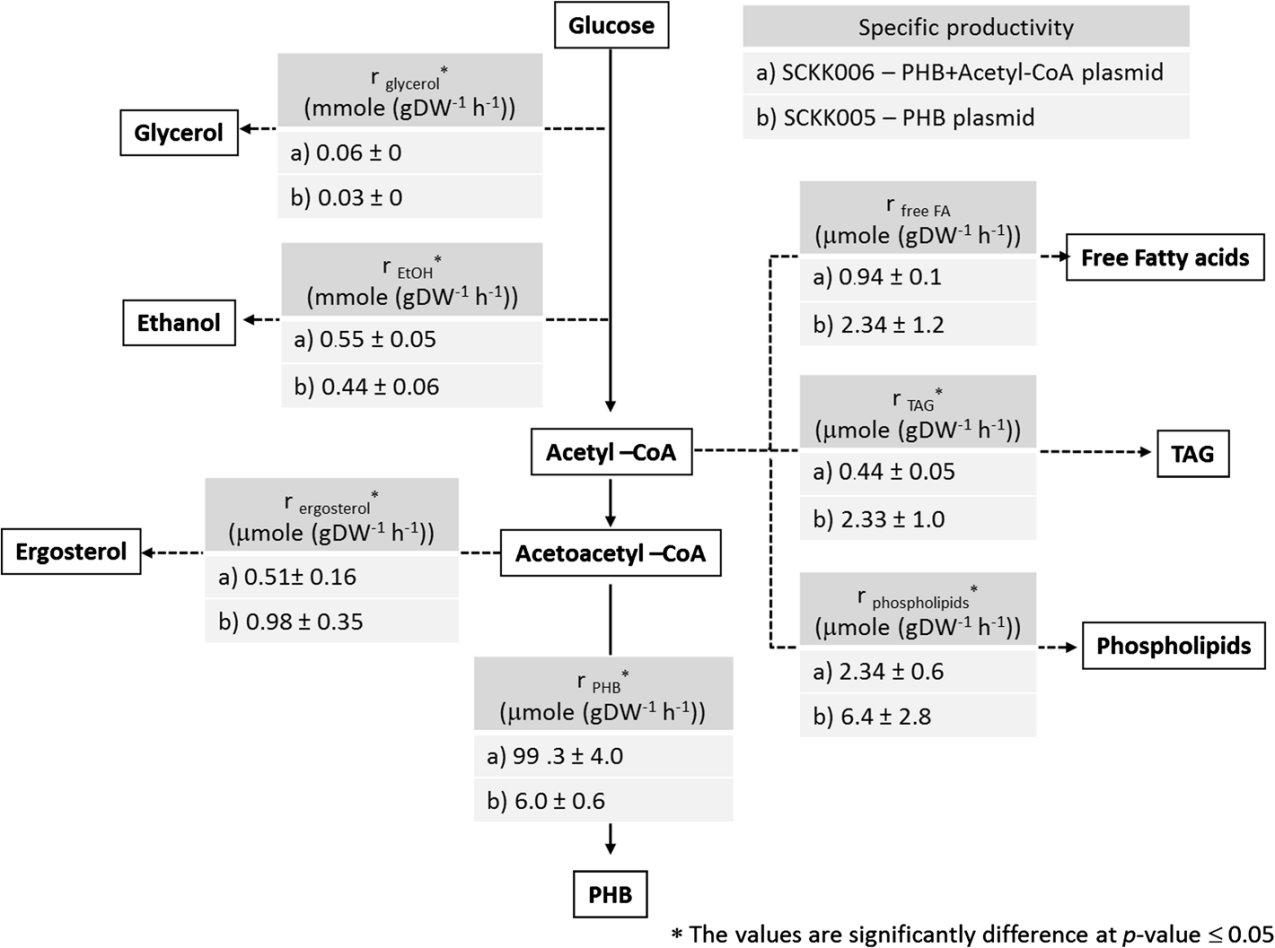
**Comparison of specific fluxes in SCKK006 and SCKK005 during growth on glucose in aerobic batch bioreactor cultivation with 20 g L**^**-1**^**glucose as carbon source.** SCKK006 is *S. cerevisiae* harboring an acetyl-CoA boost plasmid (pIYC08) and the PHB plasmid (pKK01). SCKK005 is *S. cerevisiae* harboring an empty plasmid (pIYC04) and the PHB plasmid (pKK01). The fluxes towards the different lipids were calculated from measurement of the lipid composition of the biomass and the maximum specific growth rate. The mean value ± SD from at least triplicate fermentations are reported.

To further explore carbon channeling from glucose to PHB and use of acetyl-CoA for lipid synthesis we measured phospholipids, triacylglycerols, free fatty acids and ergosterol in the cells. Using these measurements we calculated the specific productivities (r_p_) of ethanol, glycerol, PHB, triacylglycerols (TAG), ergosterol, phospholipids and free fatty acids as directly and non-directly acetyl-CoA derived products during growth on glucose using the maximum specific growth rate (μ_max_) and the product yield coefficient. The biomass yield coefficient (Y_sx_) and the product yield coefficient (Y_sp_) are defined as the amount of substrate (s) consumed for the formation of biomass (x) or other metabolites (p) such as ethanol, acetate or PHB. The specific product formation rate was calculated by using the equation: $r_{p}=\mu_{\max}\cdot \frac{Y_{\mathrm{sp}}}{Y_{\mathrm{sx}}}$ and the specific glucose consumption rate was calculated by using the equation: $r_{s}=\frac{\mu_{\max}}{Y_{\mathrm{sx}}}$. Figure 5 illustrates a simplified pathway with the calculated fluxes during growth on glucose. The mean value ± SD from at least triplicate fermentations is reported. The carbon from glucose was mainly directed to ethanol as the major product in *S. cerevisiae*. The impact of using the acetyl-CoA boost plasmid was clearly associated with a higher specific productivity of PHB in SCKK006 as well as the lower specific productivities of triacylglycerol and phospholipids as other acetyl-CoA derived products. The higher specific productivity of glycerol in SCKK006 revealed the collateral effect of *ADH2* over-expression as mentioned above. The specific productivity of ergosterol, which is also derived from acetoacetyl-CoA, an intermediate in the PHB pathway, was lower in SCKK006 than in SCKK005, which shows that the heterologous PHB pathway is clearly able to compete for acetoacetyl-CoA otherwise used for biosynthesis of ergosterol. Finally, as a result of employing the acetyl-CoA boost plasmid to channel carbon from ethanol to the PHB pathway, the specific productivity of PHB in the glucose phase in the strain carrying the acetyl-CoA boost plasmid (SCKK006) was 99.3 ± 4 μmole (g DW^-1^ h^-1^), 16.5 times higher compared to the strain carrying the empty acetyl-CoA plasmid (SCKK005).

## Discussion

The addition of precursor molecules to the fermentation medium has been shown to improve the formation of many biological products, e.g. supplementation with glucosamine as precursor resulted in improved hyaluronic acid production by *E. coli* (Mao et al. 2009) and supplementation with specific amino acids enhanced heterologous protein production by *S. cerevisiae* (Görgens et al. 2005). Extra feeding of acetate (0.5 g⋅L^-1^ with pH adjusted to 4.5) and panthothenate (1000 μg⋅L^-1^) as a precursors of acetyl-CoA were tested by Carlson and Srienc in order to increase PHB accumulation in *S. cerevisiae* (Carlson and Srienc 2006). Feeding a combination of acetate and pantothenate was found to improve PHB production by approximately 45% over the control. Instead of feeding precursors to improve the productivity, which would increase the costs of PHB production, we focused on pathway engineering to increase the supply of the precursor for PHB production and hereby improve the economic feasibility of a process using *S. cerevisiae*.

The native *ADH2* in *S. cerevisiae* is a glucose-repressible alcohol dehydrogenase. It catalyzes the oxidation of ethanol to acetaldehyde only when glucose becomes depleted from the medium. In order to activate the reaction catalyzed by *ADH2* when glucose was completely consumed, the *HXT7* promoter was selected to control *ADH2* in the acetyl-CoA plasmid. Although the *HXT7* promoter is a glucose-repressible promoter, we select the *HXT7* promoter based on its strong expression level especially during the ethanol phase. Therefore, *ADH2* under control of the *HXT7* promoter ensures the conversion of ethanol to acetyldehyde and is responsible for the initial step in the utilization of ethanol as carbon source. *ALD6* encoding cytosolic aldehyde dehydrogenase is involved in the conversion of acetaldehyde to acetate. This enzyme utilizes NADP^+^ as the preferred coenzyme. As a result, *ALD6* over-expression might help providing NADPH, a required cofactor for PHB production (Boubekeur et al. 2001). There are two acetyl-CoA synthetase in *S. cerevisiae*, encoded by *ACS1* and *ACS2*. Both of these enzymes catalyze ATP-dependent activation of acetate to acetyl-CoA. The expression of *ACS1* is subjected to glucose repression while *ACS2* can be expressed during growth on glucose where it is likely to be responsible for cytosolic acetyl-CoA production. However, the regulation of both enzymes is complex and hence over-expression is believed necessary to increase the flux towards acetyl-CoA. In the native host *Salmonella enterica,* mutations at Leu-641 of acetyl CoA synthetase (Acs), prevent the acetylation by acetyltransferase bypassing the need for sirtuin deacetylase activity and maintain the enzyme in an active state during growth on acetate. (Starai et al. 2005). Enhancing acetyl-CoA supply by engineering the pyruvate dehydrogenase bypass through over-expression of acetaldehyde dehydrogenase in combination with introduction of a *S. enterica* acetyl CoA synthetase variant (L641P) in *S. cerevisiae*, was successful demonstrated to improve the productivity of isoprenoids in yeast (Shiba et al. 2007).

*CIT2*, the peroxisomal citrate synthase and *MLS1*, the cytosolic malate synthase are key enzymes of the glyoxylate shunt and hereby affect acetate metabolism (Kunze et al. 2006; Lee et al. 2011). Deletion of *CIT2* and *MLS1* in order to improve the availability of cytosolic acetyl-CoA was investigated for its impact on PHB production. However, this deletion strategy resulted in the impaired metabolism due to the incapability to utilize C_2_ carbon via the glyoxylate shunt in *cit2Δ* and *mls1Δ* strains. The deletion strains have only the tricarboxylic acid cycle (TCA cycle) to utilize C_2_ carbon for energy production whereas the biosynthesis of C_4_ dicarboxylic acids required as precursors for amino acids biosynthesis cannot take place, resulting in lack of growth (Chen et al. 2012). This is reflected by a much reduced biomass yield in the *cit2Δ* strain. In the *mls1Δ* strain (SCKK009), less acetate was accumulated in the medium compared to the *cit2Δ* strain and acetate was slowly consumed until the end of fermentation. This might be due to the activity of the homolog malate synthase encoded by *DAL7* (Hartig et al. 1992). Moreover, the deletion of *CIT2* affect acetate metabolism and reduced the efficiency of acetyl-CoA synthetase, both native acetyl-CoA synthetase and the additional acetyl-CoA synthetase, to catalyze the ATP-dependent activation of acetate to acetyl-CoA thus resulting in more than 6.5 g/L^-1^ of acetate accumulated in the *cit2Δ* strain and no further biomass production after glucose is completely consumed (after 24 hr). Besides that, the PHB titer was drastically decreased as compared to the non-deletion strain, SCKK006.

The comparison of the specific productivities in the PHB producing strains revealed that employing the acetyl-CoA boost plasmid helps in directing carbon towards the PHB pathway. Although, there was a difference in PHB yield when the PHB strains, SCKK005 and SCKK006, were cultivated in bioreactors. This might be explained by the cultivation condition in the bioreactor which favors the growth of *S. cerevisiae* rather than PHB production. Thus, the maximum specific growth rate in shake flasks was 0.18-0.20 h^-1^ while the maximum specific growth rate in the bioreactors was 0.27-0.28 h^-1^. Although the PHB yield from the bioreactors was less than shake flask cultivation the difference in PHB productivity between SCKK005 and SCKK006 clearly showed the effect of employing the acetyl-CoA plasmid for PHB production. Therefore, the flux from glucose to PHB was analyzed by using the information from aerobic batch bioreactor cultivations. For this analysis free fatty acids, TAG, phospholipids and ergosterol were considered as side products of PHB production. From this analysis we found that over-expression of *ERG10* together with *phaA* significantly reduced the specific productivities of TAG and phospholipids as side products compared to the reference strain which does not have over-expression of *ERG10*. Although increased production of acetyl-CoA might lead to fatty acid or lipid synthesis, our study show that when the cells contain a heterologous pathway to PHB flux is actually directed towards PHB rather than towards lipids. Thus, we can conclude that enhancement of acetyl-CoA production by co-expression of genes on the acetyl-CoA boost plasmid improved the productivity of PHB during growth on glucose and further enhanced the productivity of PHB approximately 16.5 times bioreactor cultivations and reduce the flux from acetyl-CoA to lipids.

## Competing interests

The authors declare that they have no competing interest.

## Authors’ contributions

JN and KK participated in the design of the study. JN and VS supervised the project and edited manuscript. YC contributed the plasmid, pIYC08, used in this study. KK performed the experimental work, analyzed the data and wrote the manuscript. All authors read and approved the final manuscript.
